# Case Report: Durable Response to the Combination of Brigatinib and Cetuximab Plus Icotinib in a NSCLC Patient Harboring EGFR L858R-T790M-cis-G796S and L718Q Resistance Mutations Following Progression With Osimertinib

**DOI:** 10.3389/fonc.2022.875313

**Published:** 2022-04-21

**Authors:** Yubo Wang, Rui Han, Mengxiao Zhu, Tingting He, Yong He

**Affiliations:** Department of Respiratory Medicine, Daping Hospital, Army Medical University, Chongqing, China

**Keywords:** brigatinib, cetuximab, T790M-cis-G796S, NSCLC, osimertinib resistance

## Abstract

The efficacy of osimertinib is severely limited by the emergence of EGFR C797S, which is detected in either the cis or trans position with T790M when osimertinib is used as a second-line treatment, and which is largely identified in combination with an EGFR 19 deletion. The EGFR T790M-cis-G796S mutation, which also occurs in exon 20 as C797S, participates in osimertinib resistance. To date, limited data for overcoming this resistance mutation have been reported. Here, we report data for an advanced NSCLC patient who developed EGFR L858R-T790M-cis-G796S and EGFR L718Q resistance co-mutations following progression with osimertinib. Such a case has rarely been reported, and under chemotherapy guidelines for this situation, no other effective treatment is recommended. The patient in our case experienced remarkable clinical improvement and good tolerance to the combination target therapy of brigatinib and cetuximab plus icotinib. At the time of our patient’s last follow-up and prior to publication, our patient had reached more than 9 months of progression-free survival (PFS) and felt very well. Our finding provides clinical evidence that the combined target therapy of brigatinib and cetuximab may potentially be an effective treatment strategy for patients with an acquired EGFR T790M-cis-G796S resistance mutation following osimertinib treatment.

## Introduction

The third-generation, irreversible epidermal growth factor receptor-tyrosine kinase inhibitor (EGFR-TKI) osimertinib targets both the EGFR-sensitive mutation and the EGFR T790M resistance mutation following the progression of first- or second-generation EGFR-TKIs. Yet, most patients that have progressed on osimertinib have been found to have activation bypass pathways or newly acquired EGFR-resistant mutations (1). Recently, greater numbers of EGFR mutations have been determined for patients. However, the roles of these EGFR mutations in osimertinib resistance remain unknown. The EGFR L718Q and EGFR G796S mutations have been reported to participate in osimertinib resistance, although limited data related to overcoming these resistance mutations have been reported (2, 3). Since there is currently no effective treatment strategy, with the exception of chemotherapy, understanding triple EGFR mutations in cis is now an unmet need. The combination of brigatinib and cetuximab has been reported to be an effective treatment for patients who acquire EGFR T790M-cis-C797S-mediated resistance to osimertinib (4). As such, brigatinib and cetuximab may be a promising treatment strategy for triple EGFR-resistant mutations.

Here, we report the first successful case for the combined use of brigatinib, cetuximab, and icotinib as a treatment for overcoming the resistance co-mutations of L858R-T790M-cis-G796S and EGFR L718Q following progression with osimertinib.

## Case Presentation

**Figure 1:**
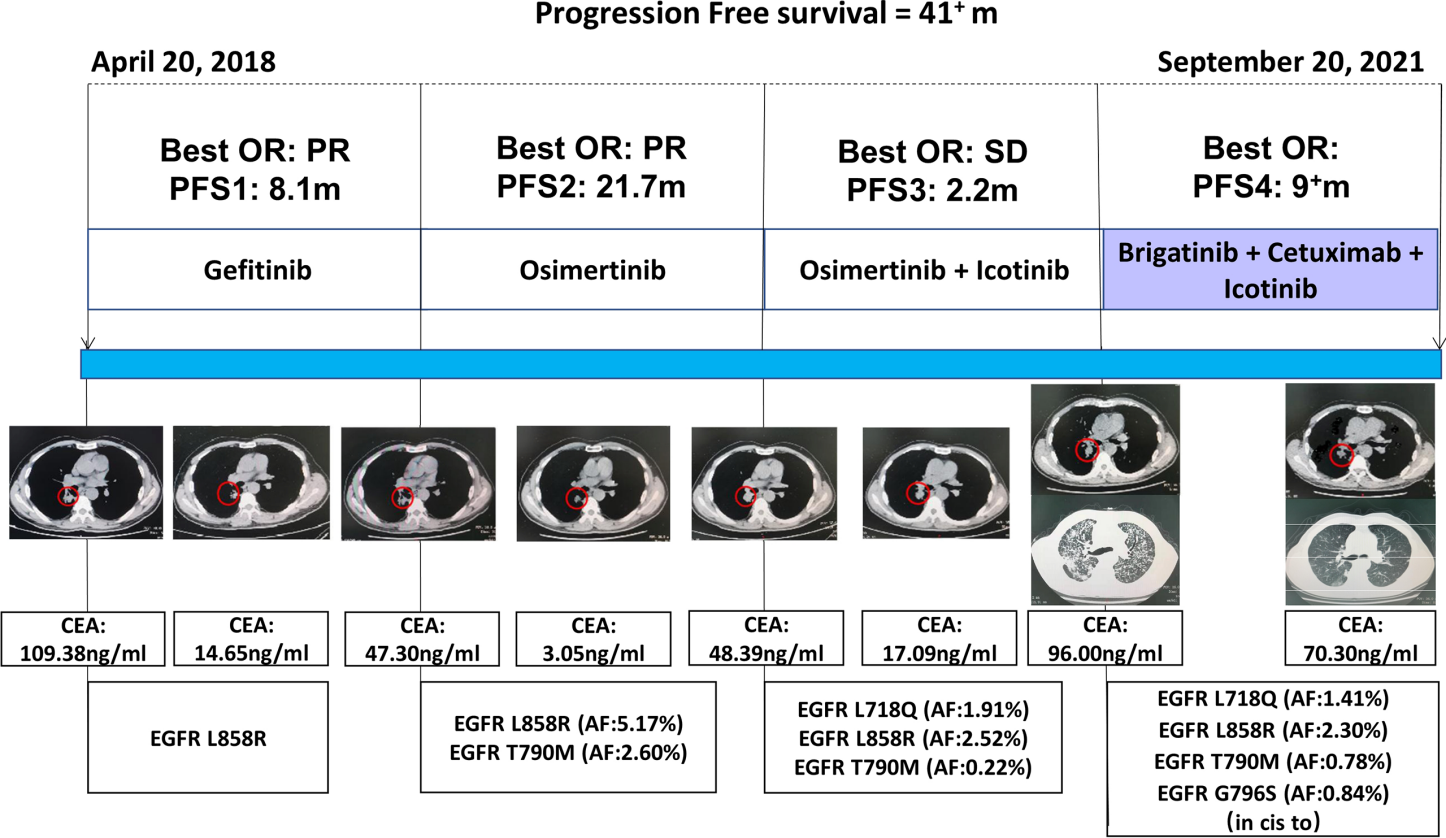
The therapeutic regimens received, the associated best objective response (OR), progression-free survival (PFS), the CEA for each line of therapy, and the major mutations detected. PR indicates a partial response. SD indicates stable disease.

In April 2018, a 61 year-old Chinese man, who was a former smoker, was diagnosed with Stage IV (T1N2M1) lung adenocarcinoma at Daping Hospital, located in Chongqing, China. Owing to the detection of an EGFR L858R mutation in tumor biopsy sample using the amplification-refractory mutation system (ARMS), the patient received gefitinib 250 mg/qd as a first-line treatment. The best objective response (OR) was a partial response (PR), with the carcinoma embryonic antigen (CEA) level decreasing from 109.38 to 14.65 ng/ml (**Figure 1**). The patient experienced an increase in aspartate aminotransferase (AST) and alanine aminotransferase (ALT) levels during treatment with gefitinib. In December 2018, 8.1 months from the time of diagnosis, the patient developed progressive disease in the lung. Liquid biopsy from plasma using next-generation sequencing (NGS) identified T790M (mutant allele frequency (MAF): 2.60%) and L858R (MAF: 5.17%) mutations. Given this outcome, osimertinib 80 mg/qd was initiated, and the best OR was PR. Unfortunately, in September 2020, the patient once again experienced disease progression following 21.7 months of osimertinib treatment, with the CEA level increasing to 48.39 ng/ml. Chest CT scans revealed an enlargement of the primary lung tumor. Liquid biopsy NGS testing from plasma indicated that the EGFR L858R (MAF: 2.52%) and T790M (MAF: 0.22%) mutations remained. A new EGFR L718Q (MAF: 1.91%) mutation additionally emerged.

L718Q has been reported to be a resistance mechanism for osimertinib, although, according to previous reports (5, 6), it may be sensitive to first- or second-generation EGFR-TKIs. Given the hepatotoxicity of gefitinib and the fact that our patient’s T790M mutation still existed, osimertinib 80 mg/qd plus icotinib 125 mg/tid was administrated beginning in October 2020. The best OR was stable disease (SD), with the CEA level decreasing to 17.09 ng/ml. Approximately 2.2 months later, the patient developed a cough and dyspnea, with the CEA level increasing to 96.00 ng/ml. Chest CT scans indicated that lymphangitic carcinomatosis had appeared. A plasma-based NGS assay was again performed and yielded a new EGFR G796S (MAF: 0.84%) mutation and a PIK3CA (MAF: 0.13%) mutation, in addition to the previously determined EGFR L858R (MAF: 2.30%), T790M (MAF: 0.78%), and L718Q (MAF: 1.41%) mutations.

For our patient, the EGFR G796S mutation, which also occurred in exon 20, existed in cis for T790M. T790M-cis-G796S has been reported to be a resistance mechanism for osimertinib and the lack of recommendations for subsequent treatment (2). Based on our previous studies, we understood that combined targeted therapy, consisting of brigatinib and cetuximab, may be an effective treatment strategy for patients with EGFR T790M-cis-C797S occurring in exon 20 and having a resistance to osimertinib (4). Therefore, since, at this point, no other targeted therapy options for EGFR T790M-cis-G796S and L718Q mutations existed given previous treatment interventions, beginning in February 2021, we decided to treat our patient with brigatinib (taken orally once daily at an initial dose of 90 mg for 7 days and increased to 180 mg from day 8) and cetuximab (500 mg/m^2^, administered intravenously on days 1 and 8 for a 21-day cycle) in combination with icotinib.

Given this new treatment plan, our patient’s cough and dyspnea were significantly and quickly relieved. A follow-up CT scan demonstrated that the lung primary lesion had obviously shrank and that the best OR was PR. Until the time of the last follow-up and at the time of publication, our patient was still responding to the brigatinib and cetuximab plus icotinib treatment, with a PFS of more than 9 months. To date, the only reported treatment side effects have been a grade II rash and grade I fatigue.

## Discussion

Resistance mechanisms for osimertinib have been investigated for a long period of time. However, some resistance mechanisms remain largely unknown. The efficacy of osimertinib as a second-line treatment is severely limited by the emergence of EGFR C797S, detected in either the cis or trans position with T790M, and mostly identified in combination with an EGFR 19 deletion. Like EGFR C797S, EGFR T790M-cis-G796S mutation also occurs in exon 20 and participates in osimertinib resistance as well. However, limited data for overcoming this resistance mutation have been reported (3). EGFR G796S in cis with T790M indicates that a combination of different generations of EGFR-TKIs are unlikely to be successful (3).

A previous case report described a patient with an EGFR L858R-T790M-cis-G796S mutation following osimertinib treatment that was enrolled in a clinical trial of pembrolizumab in combination with the oral IDO-1 inhibitor epacadostat; the patient reached PR for at least 5 months (2). A recently updated case report for the same patient indicated that the patient responded to the treatment of amivantamab (JNJ-61186372) for more than 100 days following progression with pembrolizumab (7). Although the above treatments may be effective, the accessibility of these drugs prevents clinical application. The guideline for treatment following the progression of late-line osimertinib is limited, with only local therapies and systemic therapies such as chemotherapy being recommended.

For our case, in addition to an EGFR T790M-cis-G796S resistance mutation, EGFR L718Q also still existed, which increased treatment difficulty. Due to the lack of recommendations for subsequent treatment, with the exception of chemotherapy, we employed brigatinib and cetuximab, based on our previous experience (4), in order to overcome the EGFR L858R-T790M-cis-G796S-resistant mutation. Icotinib was continued as treatment for the EGFR L718Q mutation that still existed. Our patient provided written informed consent to receive this combination therapy. Fortunately, this combination targeted therapy was successful. After receiving four lines of targeted therapy, the PFS for our patient reached more than 9 months and he was, at the time of publication, still receiving this combination treatment.

Despite the fact that the three targeted drugs are currently being taken together, tolerant toxicity has been maintained. To our knowledge, this is the first case report that provides clinical evidence that brigatinib combined with cetuximab is a promising strategy for resolving EGFR L858R-T790M-cis-G796S-resistant mutation following osimertinib progression. Although, here, we are just providing a case report, we hope our experience can still shed some light for overcoming this EGFR tertiary-resistant mutation.

## Conclusion

In this case report, we presented the first successful case of the combined use of brigatinib, cetuximab, and icotinib for overcoming the resistance co-mutations of EGFR L858R-T790M-cis-G796S and L718Q following progression with osimertinib. Our findings provide clinical evidence that the combined targeted therapy of brigatinib and cetuximab may be an effective treatment strategy for patients with an acquired EGFR T790M-cis-G796S resistance mutation following osimertinib progression.

## Data Availability Statement

The raw data supporting the conclusions of this article will be made available by the authors, without undue reservation.

## Ethics Statement

Written informed consent was obtained from the individual(s) for the publication of any potentially identifiable images or data included in this article.

## Author Contributions

YW: writing—original draft preparation. RH: data curation, software. MZ: figure preparation. TH: original data collection. YH: writing—reviewing and editing. All authors contributed to the article and approved the submitted version.

## Funding

This study was supported by funding from the National Natural Science Foundation of the People’s Republic of China (grant no. 81702291).

## Conflict of Interest

The authors declare that the research was conducted in the absence of any commercial or financial relationships that could be construed as a potential conflict of interest.

## Publisher’s Note

All claims expressed in this article are solely those of the authors and do not necessarily represent those of their affiliated organizations, or those of the publisher, the editors and the reviewers. Any product that may be evaluated in this article, or claim that may be made by its manufacturer, is not guaranteed or endorsed by the publisher.

## References

[1] Leonetti A Sharma S Minari R Perego P Giovannetti E Tiseo M. : Resistance Mechanisms to Osimertinib in EGFR-Mutated Non-Small Cell Lung Cancer. Br J Cancer (2019) 121(9):725–37. doi: 10.1038/s41416-019-0573-8 PMC6889286 31564718

[2] Klempner SJ Mehta P Schrock AB Ali SM Ou SI. Cis-Oriented Solvent-Front EGFR G796S Mutation in Tissue and Ctdna in a Patient Progressing on Osimertinib: A Case Report and Review of the Literature. Lung Cancer (Auckl) (2017) 8:241–7. doi: 10.2147/lctt.S147129 PMC5723122 29255376

[3] Ou SI Cui J Schrock AB Goldberg ME Zhu VW Albacker L. Emergence of Novel and Dominant Acquired EGFR Solvent-Front Mutations at Gly796 (G796S/R) Together With C797S/R and L792F/H Mutations in One EGFR (L858R/T790M) NSCLC Patient Who Progressed on Osimertinib. Lung Cancer (2017) 108:228–31. doi: 10.1016/j.lungcan.2017.04.003 28625641

[4] Wang Y Yang N Zhang Y Li L Han R Zhu M. Effective Treatment of Lung Adenocarcinoma Harboring EGFR-Activating Mutation, T790M, and Cis-C797S Triple Mutations by Brigatinib and Cetuximab Combination Therapy. J Thorac Oncol (2020) 15(8):1369–75. doi: 10.1016/j.jtho.2020.04.014 32353596

[5] Ma L Chen R Wang F Ma LL Yuan MM Chen RR. EGFR L718Q Mutation Occurs Without T790M Mutation in a Lung Adenocarcinoma Patient With Acquired Resistance to Osimertinib. Ann Transl Med (2019) 7(9):207. doi: 10.21037/atm.2019.04.37 31205925 PMC6545316

[6] Yang X Huang C Chen R Zhao J. Resolving Resistance to Osimertinib Therapy With Afatinib in an NSCLC Patient With EGFR L718Q Mutation. Clin Lung Cancer (2020) 21(4):e258–60. doi: 10.1016/j.cllc.2019.12.002 32146032

[7] Nagasaka M Balmanoukian AS Madison R Zhang SS Klempner SJ Ou SI. Amivantamab (JNJ-61186372) Induces Clinical, Biochemical, Molecular, and Radiographic Response in a Treatment-Refractory NSCLC Patient Harboring Amplified Triple EGFR Mutations (L858R/T790M/G796S) in Cis. Lung Cancer (2022) 164:52–5. doi: 10.1016/j.lungcan.2021.12.022 35032819

